# HIV-infected macrophages and microglia that survive acute infection become viral reservoirs by a mechanism involving Bim

**DOI:** 10.1038/s41598-017-12758-w

**Published:** 2017-10-09

**Authors:** Paul Castellano, Lisa Prevedel, Eliseo A. Eugenin

**Affiliations:** 1 Public Health Research Institute (PHRI), Newark, NJ USA; 2 Department of Microbiology, Biochemistry and Molecular Genetics, Rutgers New Jersey Medical School, Rutgers the State University of NJ, Newark, NJ USA

## Abstract

While HIV kills most of the cells it infects, a small number of infected cells survive and become latent viral reservoirs, posing a significant barrier to HIV eradication. However, the mechanism by which immune cells resist HIV-induced apoptosis is still incompletely understood. Here, we demonstrate that while acute HIV infection of human microglia/macrophages results in massive apoptosis, a small population of HIV-infected cells survive infection, silence viral replication, and can reactivate viral production upon specific treatments. We also found that HIV fusion inhibitors intended for use as antiretroviral therapies extended the survival of HIV-infected macrophages. Analysis of the pro- and anti-apoptotic pathways indicated no significant changes in Bcl-2, Mcl-1, Bak, Bax or caspase activation, suggesting that HIV blocks a very early step of apoptosis. Interestingly, Bim, a highly pro-apoptotic negative regulator of Bcl-2, was upregulated and recruited into the mitochondria in latently HIV-infected macrophages both *in vitro* and *in vivo*. Together, these results demonstrate that macrophages/microglia act as HIV reservoirs and utilize a novel mechanism to prevent HIV-induced apoptosis. Furthermore, they also suggest that Bim recruitment to mitochondria could be used as a biomarker of viral reservoirs *in vivo*.

## Introduction

A major challenge to eradicate HIV is the formation of long-lasting HIV reservoirs that are resistant to antiretroviral therapy (ART). The best recognized and examined viral reservoir corresponds to different CD4^+^ T cell populations, including naïve and memory CD4^+^ T cells. Infection of both populations has been extensively reported in HIV-infected individuals^1^. It is believed that this pool of HIV-infected cells corresponds to <0.05% of circulating cells during asymptomatic infection under effective ART^2^.

It is widely accepted that monocyte/macrophage lineage cells are among the first cells targeted by HIV^3^, and that these cells then allow the virus to spread rapidly by transmission to CD4^+^ T cells^4–6^. Macrophages are terminally differentiated, non-dividing cells derived from circulating monocytes and reside in various tissues, where they are referred to by different names, such as perivascular or alveolar macrophages, Kupffer’s cells in the liver, or microglia in the brain^7^. Under inflammatory conditions, macrophages derived from recently transmigrated monocytes die after few days^8^, whereas microglia, perivascular, and alveolar macrophages can survive for long periods – from weeks to years^9–11^. The properties of mobility, capacity for tissue infiltration, and extended survival have been proposed by several groups to be critical for the role of macrophages in the generation, stability, dissemination, and reactivation of HIV reservoirs. Furthermore, it has been demonstrated that macrophages alone can sustain HIV replication *in vivo*, supporting the hypothesis that macrophages are a primary target of HIV and may help transmit the infection to other cell types^12,13^. Nevertheless, the role of monocyte/macrophages as HIV reservoirs has been largely ignored.

Although HIV infection kills most CD4^+^ T cells, infected macrophages survive for extended periods by harboring the virus in cell membrane invaginations that protect virions from antiretroviral treatment (ART) and circulating neutralizing antibodies^14–16^. To understand how HIV promotes survival of some infected cells that ultimately become viral reservoirs, several mechanisms of HIV-mediated effects on apoptotic pathways have been examined. Several studies have demonstrated the effects of viral proteins on apoptotic protein expression including Bcl-2, Bax, FLICE inhibitory protein (cFLIP) and X-linked inhibitor of apoptosis (XIAP)^17,18^. However, most of these studies have been performed in primary and T-cell lines, while the mechanisms of extended survival and viral transmission by HIV-infected macrophages are still unknown.

Here we demonstrate that HIV-infected human microglia and macrophages function as viral reservoirs. We show that these cells survive HIV infection for extended periods of time by avoiding apoptosis and serving as long lasting viral reservoirs. We also identify that accumulation of Bim in the mitochondria of surviving HIV-infected cells does not result in apoptosis and that Bim in the mitochondria could be used as a biomarker of viral reservoirs *in vitro* and *in vivo*.

## Results

### HIV infects and induces apoptosis in human microglia, but a small population of HIV-infected microglia survives the infection

**Figure 1:**
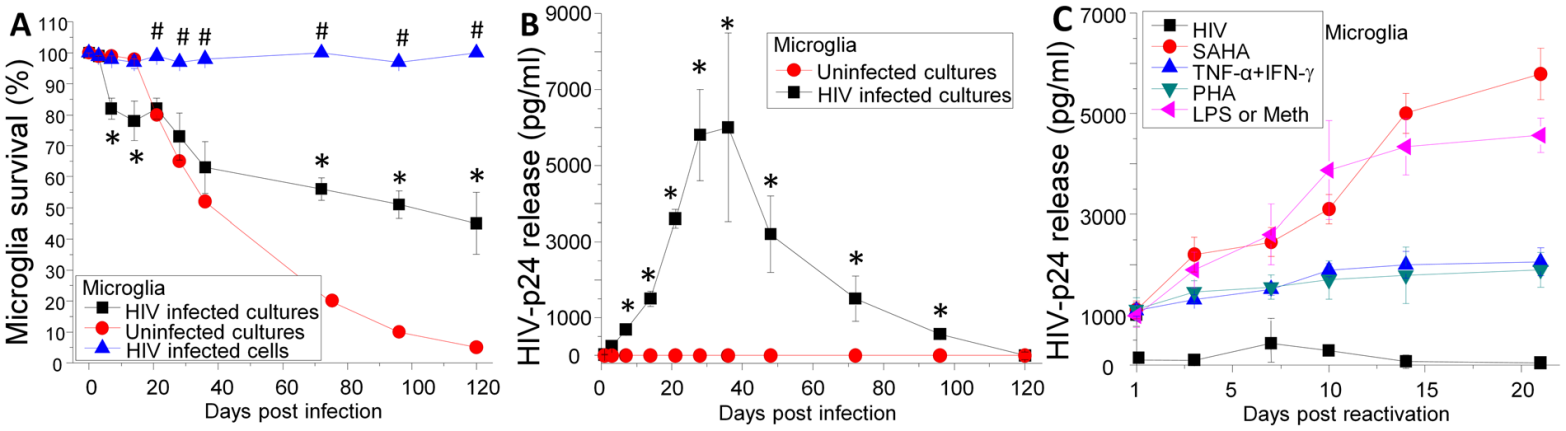
Human microglia are HIV reservoirs. (**A**) Determination of human microglia survival after HIV infection by TUNEL staining. To determine apoptosis, we perform TUNEL in combination with HIV-p24 staining and the number of positive and negative cells were quantified by microscopy. Lines with circles account for the survival of uninfected cultures which died after 21 days in culture. However, the number of apoptotic cells was reduced in HIV-infected cultures. Lines with squares represent the survival/apoptosis of HIV-infected cultures of microglia (a mix of uninfected and HIV-infected cells. HIV infected alone in the mixed culture are plotted as a line with triangles).To determine the apoptosis of just the HIV-infected microglia, we quantify apoptosis in HIV-p24 positive cells only in the mixed cultures of uninfected and HIV-infected cells. Lines with upper triangles represent apoptosis of HIV-infected cells. Minimal changes in the rate of apoptosis were detected in these cells during the time course examined (n = 3). (**B**) Time course of HIV replication in human microglia exposed to HIV_ADA_. Viral replication was measured by HIV-p24 ELISA (n = 4 different donors). Lines with circles represent uninfected cultures. Lines with squares represent HIV replication of human microglia. All points are significantly different from control conditions except by times 0 and 120 days. HIV-p24 secretion was undetectable after 120–150 days in culture. (**C**) Using the same HIV-infected microglia described in (**B**), with undetectable replication, after 120–150 days post infection, cells were treated with different factors to reactivate replication. Treatment of latently infected primary cultures of microglia results in viral reactivation. In microglia, SAHA, PHA, LPS, methamphetamine (Meth), and the combination of TNF-α plus IFN-γ results in viral reactivation as compared to control conditions (p < 0.05, n = 3).

To understand the dynamics of apoptosis in HIV-infected microglia, human fetal microglia were isolated, infected with HIV_ADA_, and stained with DAPI, TUNEL and HIV-p24 to quantify survival of uninfected and HIV-infected cells for up to 120 days. Uninfected cultures of human microglia underwent sustained apoptosis after 21 days in culture (Fig. 1A). HIV infection of microglial cultures resulted in faster initial apoptosis as compared to uninfected cultures up to 21 days (Fig. 1A, *p ≤ 0.007). However, after 21 days post infection, survival of microglial cultures remained stable and higher than uninfected cultures (Fig. 1A, *p ≤ 1.3 × 10^−4^). To determine whether uninfected or HIV-infected cells were surviving HIV infection, staining for DAPI (to observe the nuclei), phalloidin (to observe the shape of the cells), and HIV-p24 (to detect HIV infection) was performed using staining and subsequent microscopy analysis. The results showed that most surviving microglia in the HIV-infected cultures were HIV-p24 positive (Fig. 1A, 95.27 ± 4.68%) and corresponded to multinucleated cells (82.84 ± 20.09%), indicating HIV infection protects a small population of HIV-infected microglia from apoptosis.

### Surviving HIV-infected microglia become latently infected

To examine the dynamics of HIV replication within the infected microglia, we measured levels of HIV-p24 in culture supernatants by ELISA. HIV replication reached a plateau between 28 to 36 days post infection and then decayed to undetectable levels after 120 days post-infection (Fig. 1B). Although the medium was concentrated ten times using Amicon filters (50 kDa, EMD Millipore, Germany), no secreted HIV-p24 was detected by ELISA at 120 days suggesting that viral replication becomes silent (data not shown).

Analysis of the surviving cells by microscopy and FACS indicates that they maintain their macrophage phenotype including iba-1, CD14, CD68, CD11b/c, CD163, and CSF1R expression as well as their phagocytic function, cytokine secretion (TNF-α, IL-1β, and IL-6), and migratory properties in response to CCL2 as described (^19–22^ and data not shown). In conclusion, all surviving cells correspond to macrophages or microglia, are infected with HIV, and become latently infected.

### Latently HIV-infected microglia can be induced to reactivate viral replication

Critical features of viral reservoirs are extended survival, “hiding” the virus by suppressing viral replication and having the capability of reactivating the virus and spreading it to other cells^12,23–27^. To demonstrate that surviving HIV-infected microglia are latently infected, we induced HIV reactivation after 120-150 days post infection, when no HIV-p24 production is detected, using several factors known to be involved in CNS and peripheral HIV reactivation^12,24^. Next, we evaluated HIV replication by quantifying HIV-p24 secretion into the tissue culture medium for additional 21 days post treatment (Fig. 1C). Surviving latently HIV-infected microglia were treated with SAHA (N-hydroxy-N′-phenyl-octanediamide, suberoylanilide hydroxamic acid, a histone deacetylase inhibitor, 1 and 10 ng/ml), PHA (1 µg/ml), DKK1 (10 and 100 ng/ml), IL-1β (10 U/ml), methamphetamine (1 µM, Meth), LPS (1 µg/ml) or TNF-α (10 ng/ml) and/or IFN-γ (1 ng/ml). DKK1, IFN-γ and IL-1β did not reactivate viral replication (data not shown). However, SAHA, PHA, Meth, LPS and the combination of TNF-α and IFN-γ induced significant viral reactivation (Fig. 1C). In conclusion, the small microglial population that survives acute HIV infection and silences HIV replication can be induced to reactivate the virus upon specific treatments. Thus, these surviving cells fit the criteria of HIV reservoirs.

### HIV integrates into the host DNA, produces viral mRNA, replicates, and spreads among human macrophages

Due to the limited numbers of microglia isolated from brain tissue, subsequent experiments were performed using human macrophages. Despite the various manuscripts demonstrating active HIV infection and replication in macrophages^13,28–34^, it is still considered controversial that macrophages become infected by HIV mainly because macrophages can store cell-free virions within plasma membrane invaginations for at least a month^15,35–38^. Thus, to clarify whether macrophages are truly HIV infected, we analyzed HIV integration by multiple techniques, including fluorescent *in situ* hybridization (FISH) in combination with immunofluorescence, Alu-PCR, mRNA and HIV protein staining, as well as measurements of cell to cell dissemination at different time points (0, 7, 14, 21 and 28 days post-infection).

**Figure 2:**
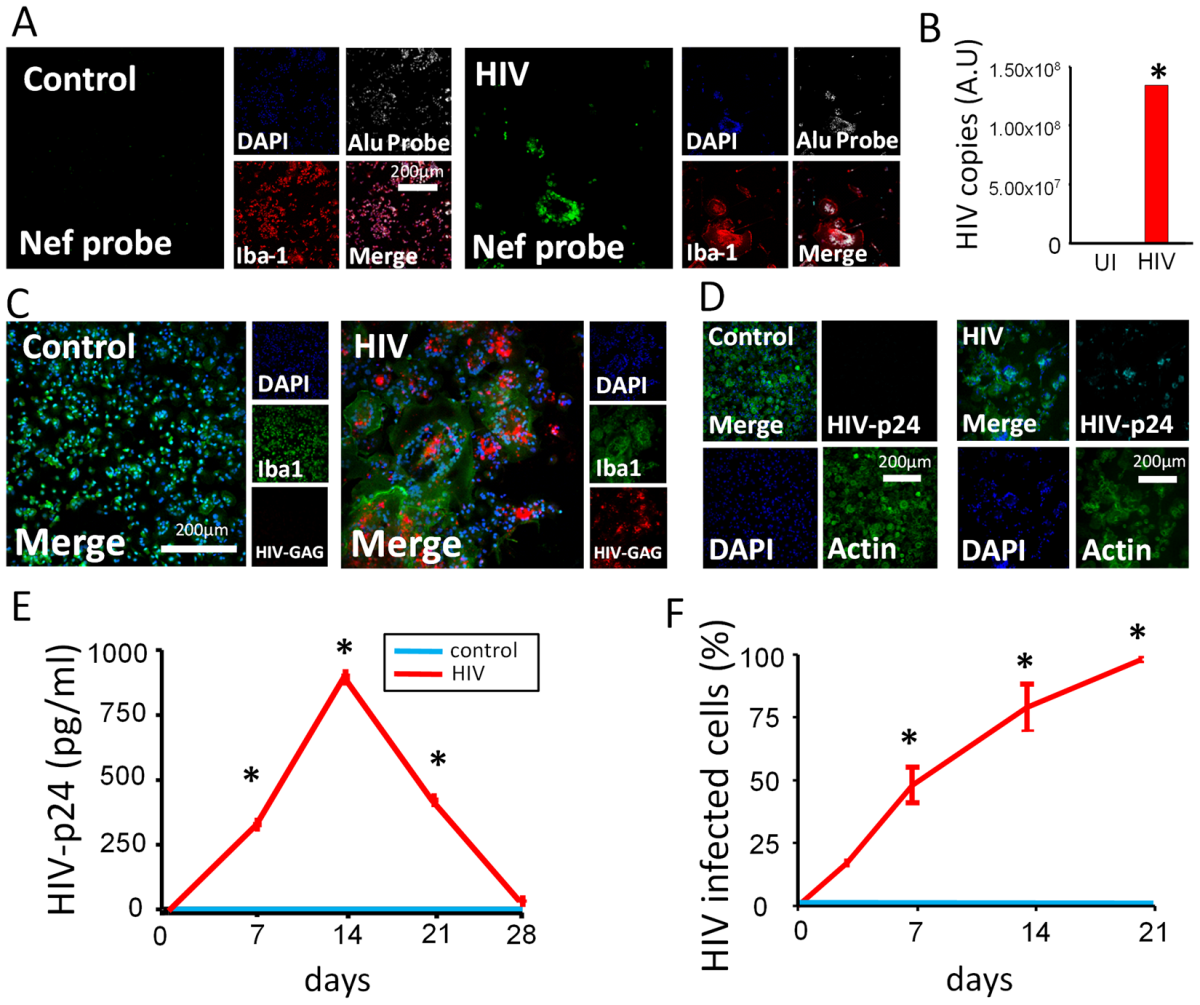
HIV infection of macrophages is productive. PBMCs were isolated by Ficoll gradient centrifugation, and macrophages were isolated by adhesion in the presence of M-CSF for 7 days. Macrophages were incubated with 50 ng/mL HIV_ADA_ and maintained in culture for further use for FISH, fluorescence microscopy, PCR, or ELISA. (**A**) A representative example of HIV-Nef DNA probe used to identify HIV DNA integration into the host DNA. A representative example of HIV DNA insertion into the host DNA after 7 days post infection with HIV_ADA_ is shown. Control (uninfected) cultures did not bind a fluorescent signal, whereas HIV treated cultures acquired the HIV DNA (green staining) colocalizing with other nuclear markers, DAPI (blue) and DNA Alu repeats (white staining). Both DNA probes (HIV-Nef and endogenous Alu) had near perfect colocalization with DAPI in HIV-infected cultures (HIV). Iba1 (red) was used as a macrophage marker, n = 3. Quantification of HIV-infection was performed by microscopy. Positive HIV-infected cells correspond to cells with Nef DNA in the nucleus with perfect colocalization with DAPI and Alu repeat probes. (**B**) Alu-gag PCR of macrophage cultures infected with HIV_ADA_ for 7 days post infection. β-globulin was used as a reference gene for fold change calculations. Alu-gag did not amplify in control (uninfected, UI) cultures (n = 3), while HIV treated cultures amplified in just over 20 cycles (n = 3). β-globulin amplified in all lysate (C_T_ = 32.46 ± 0.99, N = 6). Relative fold change calculations of Alu-GAG from control to HIV treated cultures using β-globulin as a reference gene (*p = 0.0187, n = 3). (**C**) A representative example of HIV-gag RNA probe after 7 days post infection with HIV_ADA_. Control (uninfected) cultures did not produce an HIV RNA fluorescent signal, whereas HIV treated cultures produced a fluorescent signal (red). Iba1 (green) was used as a marker for macrophages, and DAPI (blue) was used to mark nuclei. Interspersion of mRNA within subcellular locales of HIV treated cultures was unremarkable (n = 3). (**D**) A representative example of HIV-p24 antibody staining with biotin-streptavidin amplification of fluorescence after 7 days post infection with HIV_ADA_. Control (uninfected) cultures did not produce a fluorescent signal, whereas HIV treated cultures produced a fluorescent signal (cyan, HIV-p24). DAPI (blue) and actin (phalloidin, green) were used as cell markers (n = 3). (**E**) The supernatant was collected daily for 28 days post infection to determine HIV release into the extracellular media using ELISA. HIV-p24 was not detected in control (uninfected cultures) and was thus significant at 7, 14 and 21 days post-infection in cultures exposed to HIV (p ≤ 9.612 × 10^−5^, n = 6). Beyond 21 days post infection, significant levels of HIV-p24 above control were not detected. (**F**) Percentage of HIV-infected macrophages assessed by HIV-p24 staining. Macrophage cultures were fixed and stained for fluorescent microscopy using the same method described for Fig. 2D. The percentage of infected macrophages increased to significance at 3 days postinfection and continued until 100% infection at 21 days post infection (*p values ≤ 0.015. n = 5).

Using FISH in combination with immunofluorescence, we detected HIV-DNA Nef integration into the host DNA only in HIV infected cultures as early as 24 h post infection and up to 21 days post-infection (Fig. 2A, HIV), where viral replication became undetectable (Fig. 2E). No HIV-DNA Nef staining was detected in uninfected macrophages; only Alu repeats, DAPI and actin showed strong signals as expected (Fig. 2A, Control). These cultures were 99–100% positive for the macrophage marker Iba-1, indicating no T cell contamination (data not shown). Alu-gag PCR confirmed HIV integration into the host DNA after 7 days post infection (Fig. 2B). Furthermore, analysis of viral RNA expression using RNAscope indicated that HIV gag mRNA was produced in macrophages during the entire time course, while no HIV gag mRNA was detected in uninfected cultures (Fig. 2C) or using a scrambled probe (data not shown). We also analyzed the intracellular expression of HIV protein p24 (HIV-p24), by cell immunostaining and by ELISA of the culture supernatant (Fig. 2D and E, respectively). HIV infection of macrophages induced the expression and release of HIV-p24 in a time-dependent manner (Fig. 2D and E, p ≤ 0.0001, n = 3). The increase in HIV-p24 in the culture supernatant from 7 to 14 days post infection confirmed that HIV infection of macrophages was productive. After 14 days HIV replication decreased indicating that some of the HIV-infected macrophages become latently infected (Fig. 2E). Furthermore, HIV was disseminated in a time-dependent manner: the first cycles of replication only infected 8.2 to 32% of all cells (Fig. 2F, 15.69 ± 12.75%) but after 21 days post infection 100% of the cells were infected (Fig. 2F, *p ≤ 0.0001, n = 3) but HIV replication was undetected (Fig. 2E). Together, these data indicate that HIV efficiently integrates into macrophage DNA, produces viral mRNA, and expresses HIV proteins. Furthermore, the increase in HIV-p24 production in the culture supernatant, as well as the spread of infection over 21 days post infection, indicate that macrophages are productively infected upon exposure to HIV. But also our data demonstrate, like microglia, macrophages become latently infected after 21 days post infection despite that 100% of the cells have integrated HIV DNA.

### HIV infection of human primary macrophages results in massive apoptosis, but a small population of HIV-infected cells survives the infection

**Figure 3:**
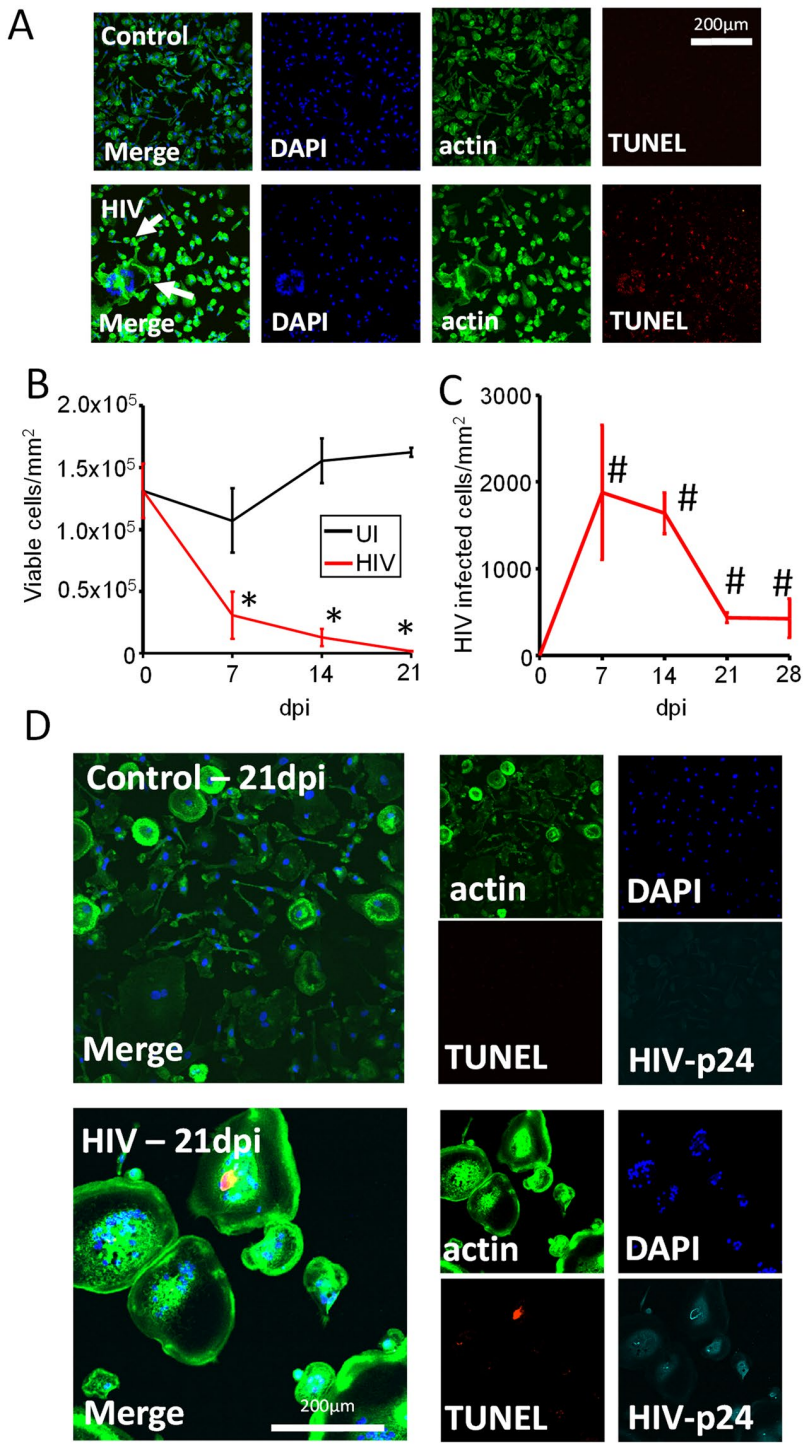
HIV infection of human primary macrophages results in massive apoptosis and survival of a small population of latently infected macrophages. Macrophages were isolated using the same methods as in Fig. 2, and cells were fixed at 7, 14, and 21 days post infection. Cells were stained to identify nuclei (DAPI, blue staining), cell shape (actin/phalloidin, green staining), and apoptosis (TUNEL, red staining). TUNEL positive macrophages were not counted as viable, with exceptions for cases of multinucleated cells. (**A**) A representative example of apoptosis in control and HIV-infected conditions after 7 days post infection. Arrows denote the presence of fused cells. (**B**) Quantification of surviving macrophages in control uninfected (UI) or HIV treated cultures (HIV) over 21 days post infection using microscopy. (**C**) Quantification of surviving HIV-infected macrophages. A significant number of HIV-infected macrophages were present at 7 and 21 days post-infection (^#^p ≤ 0.0367, n = 3 patients). (**D**) A representative example of control and HIV-infected cultures at 21 days post infection stained for nuclei (DAPI, blue), cytoskeleton (actin, phalloidin, green), apoptosis (TUNEL, red) and HIV-p24 (Cyan).

To determine whether HIV infection results in survival of some HIV-infected macrophages; cells were stained for nuclei (DAPI staining), actin (phalloidin staining), and TUNEL at different time points (0 to 21 days post infection) and we quantified the numbers of cells in 10 different fields using microscopy. Cultures of uninfected macrophages showed low levels of apoptosis up to 21 days in culture in a similar manner compared with primary microglial cultures. HIV infection of macrophage cultures resulted in a significant decrease in the total number of macrophages in the cultures, as compared to uninfected cultures (Fig. 3B, p ≤ 2.14 × 10^−7^, n = 3). A small but stable population of macrophages survived infection up to 21 days (the last point assayed; Fig. 3C). Most of the surviving cells were positive for HIV-p24 and contained HIV integrated DNA as well as showed clear signs of cell to cell fusion (Fig. 3D, HIV). HIV-infected fused macrophages have been described *in vitro* and *in vivo*, but not examined in detail, in multiple tissues even during the ART era^39–43^. In conclusion, these data indicate that similar to HIV-infected microglia, a small population of HIV-infected macrophages survive acute infection and become latently infected.

### Surviving fused HIV-infected macrophages are generated by the cell to cell fusion

**Figure 4:**
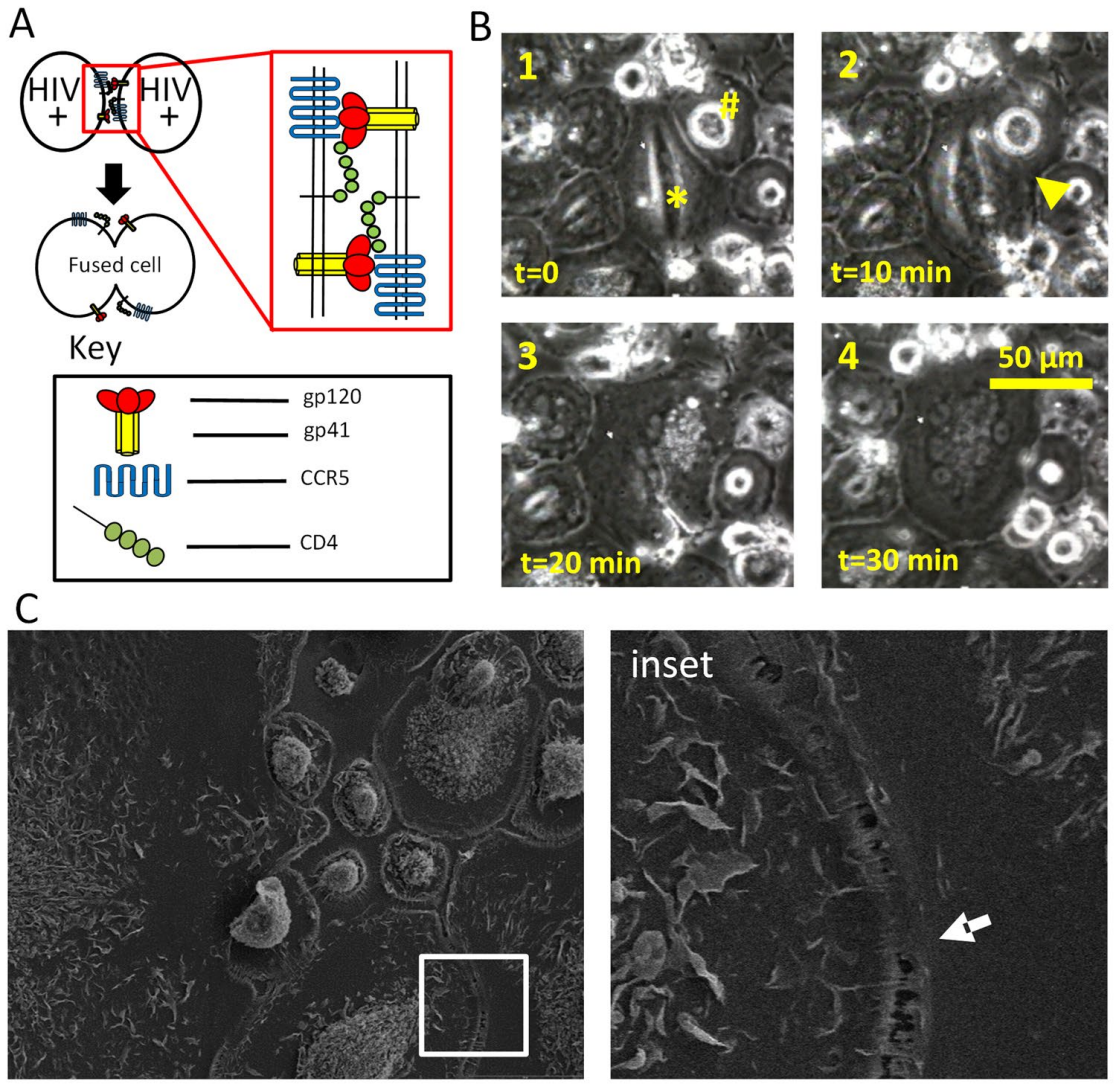
Fused HIV-infected macrophages are generated by cell-to-cell fusion and not by phagocytosis of dying cells. (**A**) Our proposed model of cell-to-cell fusion of neighboring HIV-infected macrophages. Top two circles represent neighboring macrophages infected with HIV and containing HIV Env proteins gp120/gp41 (red bulbs/yellow stalks) in proximity with endogenous CD4 and CCR5 (green bulbs, blue wave). Red outlined insert represents moment just before cell-to-cell fusion in which all components necessary are present on the neighboring cell membrane. The dark arrow represents fusion steps that result in cell-to-cell fusion, where the product of fusion is represented just below the arrow. (**B**) Representative fusion event captured by time-lapse imaging. HIV-infected macrophages were maintained in 60 mm culture dishes until cell to cell fusion was evident by light microscopy, then transferred to an incubated microscope with time-lapse capabilities. In this typical case, cell to cell fusion was captured after 3 days post infection, and fusion of two neighboring cells occurred in approximately 30 minutes. The fusion of two cells is denoted by “*” and “^#^”, and frames presented identify cellular events that are consistent with cell-to-cell fusion. Frame 1 depicts neighboring cells before fusion. Frame 2 illustrates the fusion point between cells (yellow arrow). Note the cell membrane between the two cells is nearly indistinguishable. Frame 3 illustrates the point at which the borders of the two neighboring cells are indistinguishable, with a southeastern invagination the only indicator that the new cell was once part of a pair. Frame 4 depicts the completion of the fusion event. Note that the size of the resultant cell is nearly the additive size of the original cells marked “*” and “^#^” from frame 1. (**C**) Scanning electron micrograph of melding cytoplasm between neighboring macrophages in HIV treated cultures.

Currently, they are at least four different mechanisms of multinucleation described in different diseases. First, it has been proposed that cell-to-cell fusion is not truly a result of fusion but instead correspond to phagocytosis of damaged cells^40,44^. The second mechanism is due to incomplete mitosis^40,45^; however, macrophages are terminally differentiated and do not divide. Third, cell to cell fusion by mechanisms involving IL-4, CD44, SIRP-α, macrophages fusion receptor (MFR)^46,47^ and P_2_X_7_ (see review by^48^). Fourth, a mechanism of HIV-induced cell fusion has between proposed involving gp120, gp41, CCR5, and CD4^49^ (Fig. 4A).

To test the third mechanism, we used oxidized ATP (oATP, 100 µM) to block purinergic receptors including P_2_X_7_, and probenecid (500 µM) to block pannexin and connexin hemichannels that control the release of ATP into the extracellular medium. We found that neither of these inhibitors altered cell to cell fusion in response to HIV infection (data not shown), suggesting that cell to cell fusion in HIV-infected condition is by an alternative mechanism. To determine whether the presence of multinucleated cells was the result of fusion or phagocytosis, live cell imaging of uninfected and HIV-infected cultures of macrophages was performed. No cell to cell fusion was detected in uninfected cultures (data not shown). However, exposure of macrophage cultures to HIV resulted in the significant cell-to-cell fusion as early as 2-3 days post-infection (Fig. 4B, 3 days post infection and 30 min of live cell imaging, see * and #). In contrast, using live cell imaging, no phagocytosis was detected in uninfected or HIV-infected conditions (data not shown). To further determine whether cell to cell fusion was present in our HIV-infected cultures, scanning electron microscopy of macrophages in the process of fusion was performed (Fig. 4C, inset). Our data indicate a clear continuous membrane between the two cells in the process of fusion (Fig. 4C, arrow). Together these data suggest that the generation of multinucleated cells in response to HIV infection was a product of cell to cell fusion and not an artifact of phagocytosis, ATP-mediated cell-to-cell fusion or incomplete mitosis.

### HIV fusion inhibitors decreased HIV replication but increased the survival of HIV-infected macrophages

**Figure 5:**
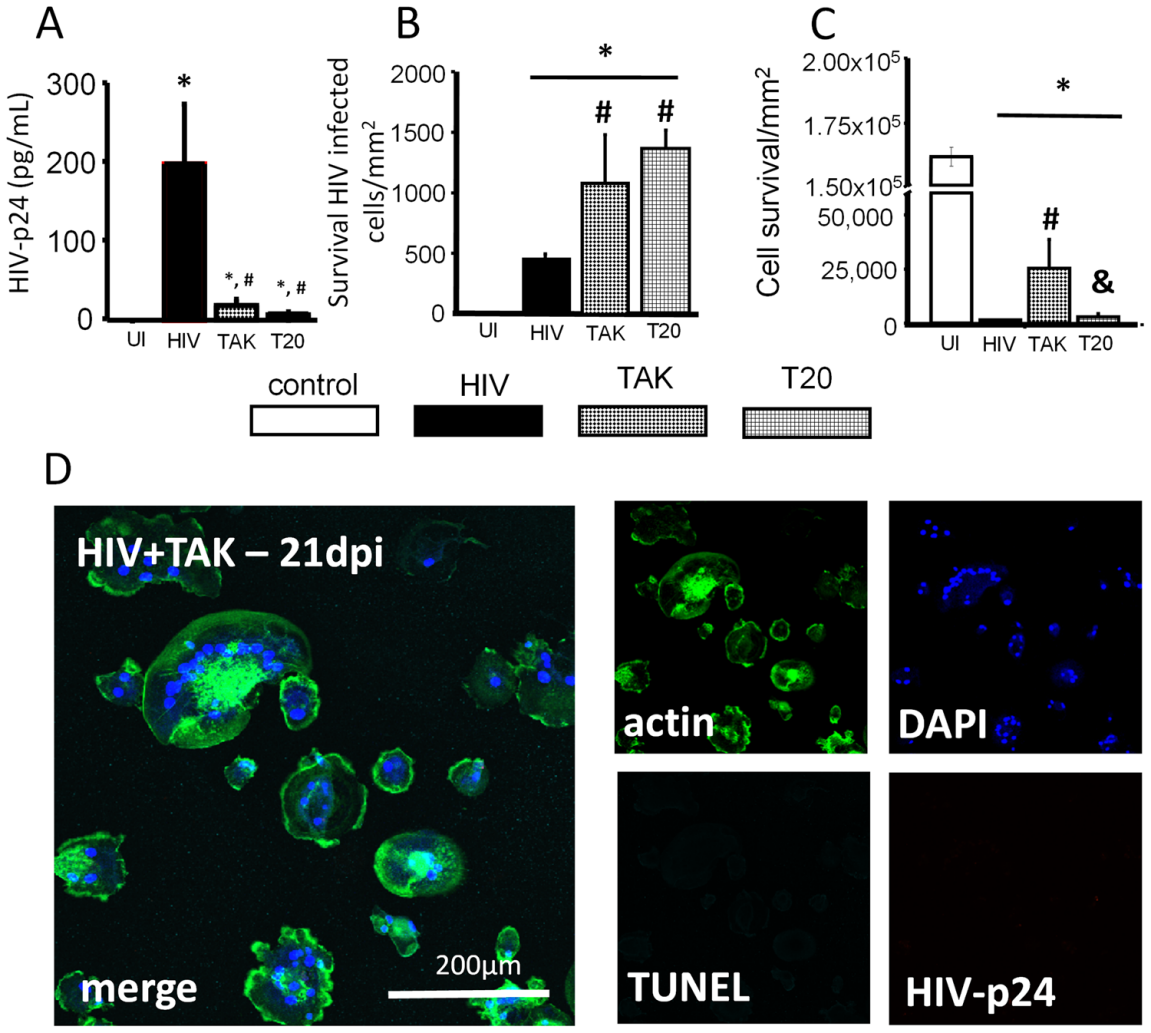
Fusion inhibitors decreased HIV replication and increased the survival of latentlyHIV infected macrophages. To assess the survival of HIV-infected macrophages in the presence of fusion inhibitors, macrophages were infected with HIV_ADA_, and fusion inhibitors were applied 24–48 hours later. This allowed for infection to establish before application of fusion inhibitors to determine whether fusion inhibitors were useful for inhibiting the formation of MGCs. The supernatant was also collected for application with ELISA. (**A**) ELISA for HIV-p24 at 21 days post infection in the presence of fusion inhibitors TAK779 (TAK), T20 or HIV alone. Fusion inhibitors TAK and T20 reduced HIV-p24 production collected from the supernatant (^#^p ≤ 0.0148 as compared to HIV alone, n = 3). Control cultures did not produce an ELISA signal above background (UI). Supernatant from HIV alone cultures contained 196.5 ± 76 pg/mL HIV-p24, a significant increase from control cultures (*p = 0.0112, n = 3). Supernatant collected from HIV infected cultures treated with TAK or T20 also contained a significant amount of HIV-p24 compared with control cultures, 16 ± 8 pg/mL, and 5 ± 2 pg/mL respectively (*p ≤ 0.0123, n = 3). The amount of HIV-p24 in the supernatant of infected cultures treated with TAK or T20 did not significantly differ from each other. The amount of HIV-p24 in supernatant from HIV alone cultures was significantly higher than both TAK779 and T20 treated cultures (^#^p ≤ 0.015, n = 3). (**B**) Fusion inhibitors prevented apoptosis of fused HIV-infected macrophages. HIV-infected cultures had significant cell death as compared to UI cultures (*p ≤ 0.0018, n = 3). Cultures treated with TAK779 or T20 contained more HIV-infected macrophages than HIV-alone cultures (^#^p = 0.0467 as compared to HIV alone, n = 3), indicating TAK and T20 treatment prevented cell death of HIV-infected macrophages over 21 days post infection, up to 28 days the last point assayed. (**C**) Quantification of non-fused cells reveals that TAK treatment improved survival of non-fused macrophages (most uninfected cells) after exposure to HIV (^#^p ≤ 0.0341; ^&^p ≤ 0.0428). T20 had no effect compared to HIV-alone cultures (n = 3). (**D**) An example of cultures treated with TAK779 (TAK) for 24 h and subsequently infected with HIV_ADA_ to demonstrate the lack of staining for HIV-p24, significant multinucleation, and cell death as compared to Fig. 3D.

To examine whether interaction between HIV-infected cells and host receptors (CD4 and CCR5) involved in cell to cell fusion participates in the survival of HIV-infected macrophages, cultures were infected with HIV for 24–48 hours to enable successful HIV integration and replication and then treated with HIV fusion blockers T20 (1 µg/mL) or TAK779 (1 µg/mL). While cell to cell fusion was not altered at any time point in the HIV-infected macrophage cultures treated with TAK779 and T20, we did observe a significant (∼99%) decrease in HIV replication (Fig. 5A). Furthermore, TAK779 and T20 treatment of HIV-infected cultures resulted in a higher numbers of HIV-infected surviving macrophages after 21 days post infection, suggesting that these fusion inhibitors increased the survival of the infected cells (Fig. 5B). TAK779 and T20 treatment also protected non-multinucleated cells present in the HIV-infected cultures from apoptosis (Fig. 5C). Microscopy analysis of the surviving cells in the HIV-infected cultures treated with TAK779 at 21 days indicates that fusion was still present, and most cells had undetectable to minimal HIV-p24 staining (Fig. 5D, HIV + TAK-21 days, compare to Fig. 3D). These results indicate that after initial infection and replication, antiretrovirals such as TAK779 and/or T20 extended the survival of latently infected cells. In conclusion, multinucleation was independent of viral production after initial infection, and HIV fusion inhibitors had an unexpected protective effect on the survival of infected macrophages.

### HIV-infected macrophages are protected from HIV-associated apoptosis

A key event in apoptosis is the formation of the transition pore in the mitochondrial membrane and the release of mitochondrial factors, such as cytochrome C, into the cytoplasm^50,51^. We have reported that HIV infection of human astrocytes results in bystander apoptosis of uninfected neighboring cells by a mechanism that involves the release of cytochrome C (CytC) into the cytoplasm^52,53^. CytC does not induce apoptosis in the infected astrocytes, but the spread of IP_3_ and calcium signals through gap junction channels causing apoptosis of uninfected neighbor cells^52,53^. It is well established that the equilibrium between expression, protein-protein interaction, and formation of the transition pore by pro-apoptotic and anti-apoptotic proteins determines the apoptotic fate of the cell^54–56^. For instance, anti-apoptotic Bcl2 protein promotes maintaining mitochondrial outer membrane pore integrity by sequestering pro-apoptotic proteins that participate in pore opening^57^. T cell lines engineered to overexpress Bcl2 have been used as a model to study latency and reactivation^58^. However, primary cells that survive infection with clinically relevant strains of HIV have never been found to contain elevated levels of Bcl2^59–61^ and if these mechanisms operate in surviving HIV-infected macrophages is unknown.

**Figure 6:**
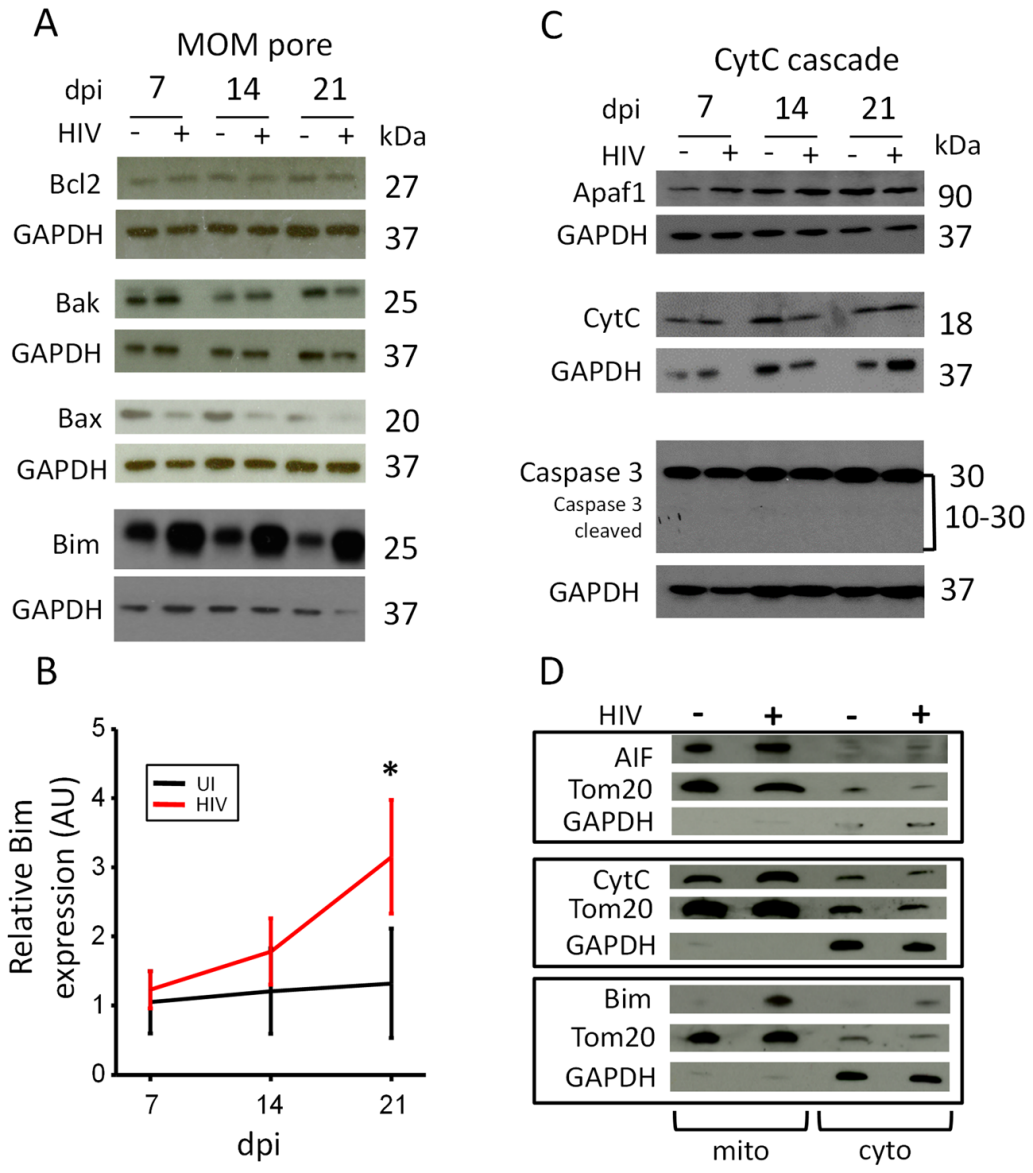
HIV-infected macrophages are protected from Bim-associated apoptosis. Macrophages were isolated and plated for the whole cell lysate collection or mitochondrial/cytoplasmic isolation for immunoblot. Cells were infected with 50 ng/mL HIV_ADA_, and the lysate was collected at 7, 14, and 21 days post infection. (**A**) Whole cell lysate was probed for pro-apoptotic and anti-apoptotic proteins that participate in MOM pore opening (anti-apoptotic: Bcl2; pro-apoptotic: Bak, Bax, Bim). There was no significant change in the expression of Bak, Bax, Bcl2, or Mcl1 (data not shown) due to HIV infection (n = 3). Pro-apoptotic protein Bim was the only protein with significant changes of expression due to HIV infection. Bim expression increased over 21 days post infection in HIV-infected macrophages when only latently HIV-infected macrophages remain in the culture. (**B**) Quantification of Bim expression in uninfected and HIV-infected macrophages. Scanning and quantification of the band intensity were performed. We observe that only after 21 days post infection, Bim was upregulated in the few surviving HIV-infected macrophages with undetectable HIV replication. (**C**) Representative immunoblot analysis of the Apaf-1, CytC, Caspase-3, and GAPDH. These essential components of the CytC apoptotic cascade apoptotic cascade are present, but not affected, during HIV infection of macrophages. Caspase 3, the final executioner caspase during CytC mediated apoptosis was not activated (cleaved) during HIV infection over 21 days post infection (n = 3). GAPDH was used as a loading control and used as a comparison protein for densitometric analysis. (**D**) Representative mitochondrial/cytoplasmic fractionation of HIV-infected cells used to identify the movement of pro-apoptotic intermembrane space content in HIV-infected macrophages compared to control cells. TOM20 was used to determine mitochondrial fractions, and GAPDH was used to determine cytoplasmic fractions. AIF or CytC presence in the cytoplasm of infected cells was not elevated during HIV infection (n = 5). However, Bim was not detected in the mitochondrial or cytoplasmic fractions of control cells but was detected in these fractions during HIV infection after 21 days post infection (*p = 0.007, n = 3).

Here we analyzed the expression of proteins involved in mitochondrial outer membrane (MOM) pore integrity and Cytochrome C-mediated apoptosis, mitochondrial fusion, and apoptosome formation including Bcl-2, Bak, Bax, Bim, mfn-1, XIAP, apaf-1, mcl-1, hsp70, mfn2, hsp27, AIF, and caspase -3 and -9. No changes in protein levels or protein cleavage of these protein was detected (data not shown and Fig. 6A). The only protein affected was Bim. We found that only Bim expression was upregulated in HIV-infected macrophage cultures after 21 days post-infection, or the time when only a few latently HIV-infected macrophages remain in culture (Fig. 6A and B). In contrast, Bim expression was stable in uninfected cultures (Fig. 6B). Also, no changes in the three Bim isoforms (EL, L, and S) were detected in our cultured macrophages (data not shown) aside of the overall increased expression. These multiple Bim isoforms are generated by alternative splicing and have been proposed to have different functions^62^. Thus, trafficking of the protein into the mitochondria or protein-protein interaction with other apoptotic proteins may be affected. Because Bim is a highly pro-apoptotic protein^63^, the observation that Bim expression was increased in the few surviving HIV-infected macrophages (Fig. 6B) suggests that Bim may have an alternative function in these cells or HIV blocks its apoptotic function.

### HIV prevents initiation of apoptosis and formation of the apoptosome

To examine the mechanisms by which HIV prevents apoptosis in a small population of HIV-infected macrophages, we examined protein expression levels of Apaf-1, CytC, and caspase-3. We detected no changes in expression or activation of caspase-3 in response to HIV infection and no changes in the molecular weights of apoptotic proteins that would result from caspase-mediated cleavage or complex formation with other proteins (Fig. 6C). Also, to identify whether apoptosis-inducing factor (AIF) or cytochrome C are released from the mitochondria into the cytoplasm, cell fractionation was performed in uninfected, and HIV-infected macrophages after 21 days post infection. Our results indicate that AIF and CytC are retained inside of the mitochondria (Fig. 6D). Thus, the apoptotic process is blocked. Finally, to determine whether the mitochondria of the surviving HIV-infected macrophages are functional, we used mitotracker, a membrane potential-sensitive dye. No changes in mitochondrial membrane potential were detected in the surviving HIV-infected macrophages as compared to uninfected cells, indicating that mitochondria are still functional (data not shown). Together, these results indicate that apoptosis is not initiated in HIV-infected macrophages that survive acute HIV infection.

### Increased Bim mitochondrial association in HIV-infected macrophages and microglia *in vitro* and *in vivo*

Bim is a highly pro-apoptotic protein in most cell types^63^ that is nevertheless highly upregulated in surviving HIV-infected macrophages where apoptosis is blocked (Fig. 6B). Normally Bim associates with the cytoskeleton, but upon apoptosis activation, Bim is recruited into the mitochondrial membrane to trigger apoptosis^64^. Using cell fractionation experiments, we determined that bim was also recruited or sequestered into the mitochondria specifically in surviving latently HIV-infected macrophages (Fig. 6D, mito fraction, HIV+). To examine whether Bim also associated with mitochondria *in vivo*, we used human tissues (lymph nodes and brains) obtained from uninfected and HIV-infected individuals without detectable viral replication and performed immunostaining for Bim, VDAC (a mitochondrial protein), HIV-p24, and DAPI.

**Figure 7:**
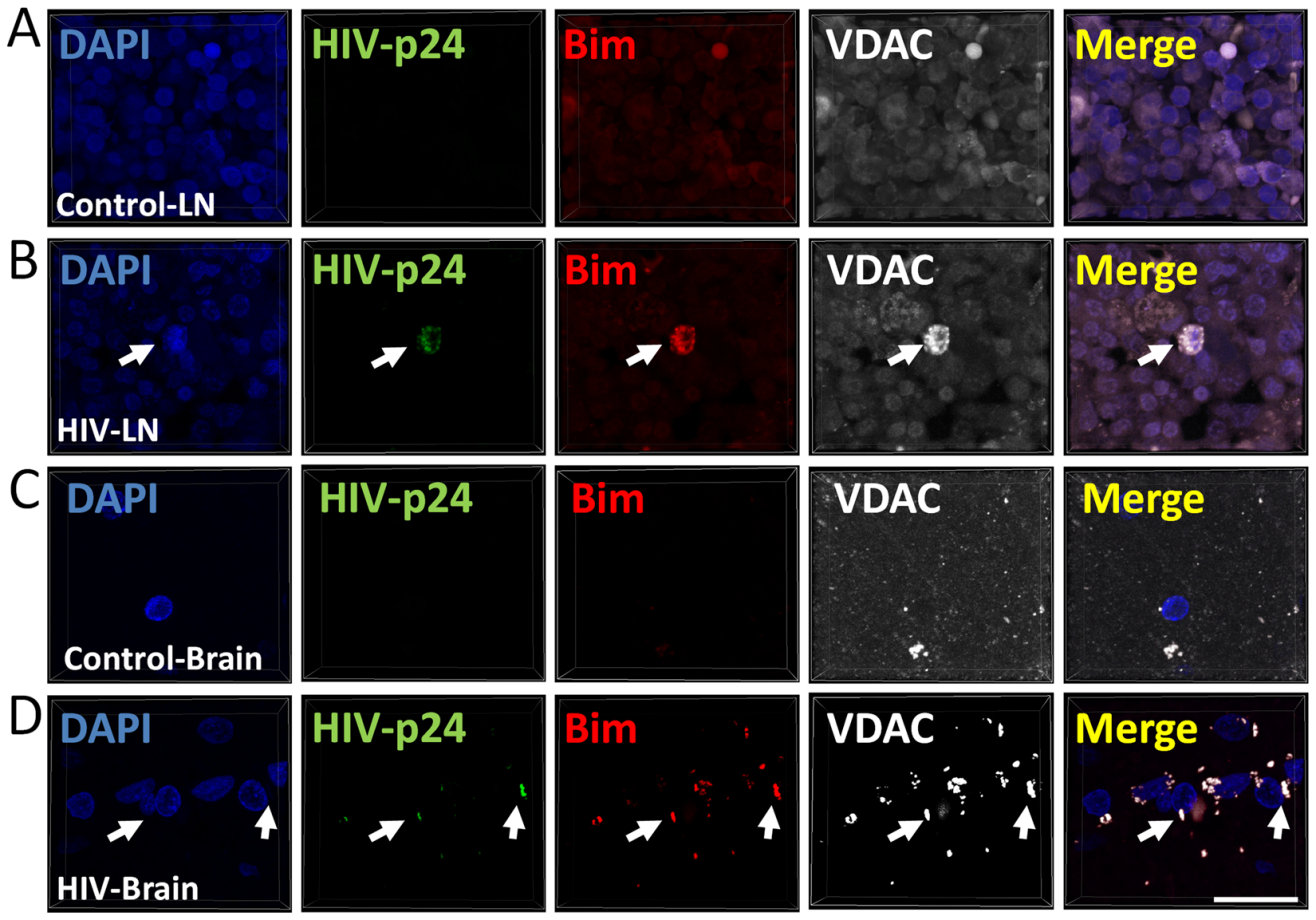
Bim is expressed in HIV reservoirs in the lymph nodes and the brain. Human lymph node and brain tissue section obtained from uninfected and HIV-infected individuals without detectable plasma viral load for several years were subjected to confocal and 3D reconstruction. Tissue was stained for Nuclei (DAPI, blue staining), Bim (red staining), VDAC (a mitochondrial marker, white staining) and HIV-p24 (HIV, green staining). Uninfected tissue sections showed little and uniform bim and VDAC staining. No staining for HIV-p24 was detected in uninfected tissue samples. Human tissue sections of lymph node (**A** and **B**) and brain (**C** and **D**) obtained from uninfected (**A** and **C**) and HIV-infected individuals (**B** and **D**) without replication detected by years (6–24 years). (**A** and **B**) Correspond to lymph tissue, mostly HIV-infected T cells, the proportion of HIV-positive cells was 1 cell per 10^8^ to 10^12^. (**C** and **D**) Correspond to human brain tissue sections obtained from uninfected and HIV-infected individuals. Most HIV positive cells correspond to macrophage/microglia. There are not lymphocytes in the CNS. Similar rates of HIV-positive cells were detected in brain tissue. Images from HIV-infected tissues correspond to the few HIV-infected cells detected in these tissues, but all HIV positive cells had bim accumulated in the mitochondria. Bar: 12 µm.

As expected, analysis of uninfected lymph nodes and brains showed no staining for HIV-p24 protein (Fig. 7, control-LN and control brain, A and C). Furthermore, in uninfected tissues diffuse Bim and VDAC staining was observed in lymph nodes (Fig. 7A) and brains (Fig. 7C). In contrast, in HIV-infected tissues obtained from individuals on effective ART at the time of death (6-24 years of AR treatment), a few low expressing HIV-p24 infected cells were detected (Fig. 7, HIV-LN and HIV-brain, B and C). These infected cells showed increased expression of Bim in perfect colocalization with VDAC, a mitochondrial protein (Fig. 7B and D), consistent with our *in vitro* data indicating Bim accumulates in mitochondria of HIV-infected cells *in vivo*. Our results, *in vivo*, show that both T cells and macrophages have a similar profile of low expression of HIV proteins and Bim recruited into mitochondria (1 positive cell in 10^8^ to 10^12^ cells analyzed). Thus, both cell types have a similar profile of Bim. Negative controls using IgGs and control sera did not show any staining (data not shown). Together, these data indicate that Bim expression is increased and it is recruited to the mitochondria in surviving HIV-infected macrophages/microglia, suggesting that Bim may be used as a biomarker to identify HIV reservoirs *in vivo*.

## Discussion

The data presented here demonstrate that a small population of human microglia and macrophages survive acute HIV infection, that these surviving cells are HIV-infected but protected from apoptosis, and that HIV replication is silenced but can be reactivated in these cells. This indicates that these cells act as viral reservoirs. We found that formation of these reservoirs is associated with macrophage fusion and that cell fusion inhibitors extend the survival of HIV-infected cells. Furthermore, we found that while apoptosis is blocked at an early step, the proapoptotic protein Bim is upregulated and recruited into the mitochondria in latently infected macrophages both *in vitro* and *in vivo*. Thus, we propose that Bim association with the mitochondria is a potential biomarker of latently HIV-infected macrophages/microglia *in vivo*.

The presence of fused macrophages, also known as multinucleated giant cells (MNGC), has been reported extensively in the context of AIDS^65^. Furthermore, several studies of non-AIDS conditions indicate the presence of fused cells in lymphoid organs^66–71^, HIV-associated lymphoepithelial cysts of the parotid gland^71^, and colonic mucosa^72^. Thus, these fused cells are present *in vivo*, even during effective ART, and are found in virally advantageous regions that are part of or are in close contact with lymphoid areas that can support viral reservoirs and reactivation.

Also, we found that HIV fusion inhibitors T20 and TAK779 unexpectedly resulted in extended survival of a subpopulation of uninfected and HIV-infected macrophages, probably due to the reduction in HIV replication (Fig. 2E). These results indicate that some kinds of ART, i.e. those that prevent cell fusion, may extend the survival of HIV reservoirs by avoiding the toxic effects of high replication. Our hypothesis involves two different mechanisms of cell protection induced by TAK779 and T20. First, a protective effect of these antiviral drugs in latently HIV-infected cells mediated by prevention of cell death of HIV-infected cells as described in the results, but there may be a second mechanism of cell protection of uninfected cells, by preventing binding of the virus to CD4 and CCR5. A similar mechanism of cell death protection mediated by CCR5 has been described in several cell types^73–76^. This point requires further exploration because this indicates that some ART may have negative effects on the survival of HIV reservoirs.

Although the HIV genome does not encode any apoptosis inhibitor proteins, several HIV proteins such as Tat can upregulate host anti-apoptotic proteins including Bcl-2, FLIP, XIAP and C-IAP2^77–80^. Also, HIV-Nef induces phosphorylation and inactivation of Bad, suggesting that alterations in the apoptotic process contribute to the survival of HIV-infected T cells^81^. However, we did not detect changes in expression of these apoptotic proteins in our latently HIV-infected macrophages. However, we cannot discard changes in protein-protein interactions, conformational changes, post-transcriptional modifications, or activity of the transition pore on the mitochondria altered by HIV infection. These studies will be the focus of future research. However, several laboratories have identified that the formation of transition pore is affected in other cell types, because overexpression or antagonist of Bcl-2 can control HIV replication, reactivation, susceptibility to apoptosis, but the mechanism is unclear^82–86^.

Also, our previous studies of human astrocytes indicate that in infected astrocytes the transition pore in the mitochondrial membrane is formed, and CytC is secreted from the mitochondria into the cytoplasm. HIV, however, blocks the subsequent formation of the apoptosome^87–90^. In contrast, we did not detect any evidence for the formation of the transition pore or secretion of mitochondrial factors into the cytoplasm in latently infected macrophages, indicating that HIV blocks formation or the function of the transition pore early during the apoptotic process. Thus, the mechanism of how HIV promotes the survival of HIV- infected microglia and macrophages is different from those operating in T cells and astrocytes.

A unique feature of surviving latently infected macrophages is the significant upregulation and recruitment of proapoptotic protein Bim into the mitochondria (Fig. 6B). Bim downregulation in cancer cells is related to extended survival, metastasis and improved response to cytotoxic agents (reviewed in^91,92^). We propose that upregulation and sequestration of Bim into the mitochondria in surviving infected macrophages reflects an early step in the apoptotic pathway, which is subsequently blocked by HIV because Bim recruitment is not associated with apoptosome formation, secretion of CytC or AIF into the cytoplasm, or activation of caspases. The upregulation of Bim is currently used as a promising cancer therapeutic because upregulation of this protein can result in apoptosis^63^. However, in latently HIV infected cells *in vitro* and *in vivo*, Bim did not lead to apoptosis despite its recruitment into the mitochondria. Also, as indicated in the result section, there are no changes in expression or ratios the three different isoforms of Bim. Thus, the mechanism by which Bim is blocking apoptosis of latently HIV-infected macrophages could be related to changes in host protein-protein interaction, the opening of the transition pore, or direct binding of HIV proteins to the pore. Clearly, HIV is altering mitochondrial function/signaling/metabolism^93,94^, but the interplay between metabolism, survival and viral silencing/reactivation is totally unknown and currently is under active investigation in several laboratories.

An additional explanation of the role of Bim in the surviving HIV infected macrophages is its participation in the metabolism of viral reservoirs. Survival of HIV infected macrophages results in mitochondrial fusion and changes in cell metabolism to promote survival of these cells including the use of alternative sources of fuel such as amino acids. Thus, we propose that bim plays a role in metabolic regulation of the survival of these infected cells. Interesting, Bim during the formation of the transition pore also can interact indirectly with enzymes involved in mitochondrial metabolism such as creatine kinase, and hexokinase^95^. Furthermore, both enzymes participate in HIV replication and viral reservoir formation^93,96^, but whether both set of proteins could regulate HIV reservoir metabolism is still under active investigation.

In summary, we have obtained compelling evidence that macrophages/microglia function as HIV reservoirs, and that these viral reservoirs are formed by a novel mechanism involving Bim upregulation and recruitment to mitochondria, which may be useful as a biomarker of viral reservoirs *in vivo*. These insights, together with the observation that fusion inhibitors increase the size of the latently HIV-infected macrophage pools, need to be incorporated into the current HIV reservoir paradigms and considered during the ongoing efforts to achieve HIV eradication.

## Materials and Methods

### Reagents

All reagents were purchased from Sigma (St. Louis, MO) except in the places that are indicated otherwise. HIV_ADA_, TAK779, T20, soluble CD4 (sCD4), were from the NIH AIDS Research and Reference Reagent Program (Germantown, MD). Primers and probes were obtained from Biosynthesis (Lewisville, TX) and PNAbio (Newbury Park, CA). Medium, penicillin/streptomycin (P/S), dyes and secondary antibodies were obtained from Thermo-Fisher (Waltham, MA). Human AB serum and FBS were from Lonza (Walkersville, MD). HEPES was from USB (Cleveland, OH). HIV-p24 ELISA was obtained from Perkin-Elmer (Waltham, MA). Antibodies to HIV-p24 were obtained from Genetex (Irvine, CA). All other antibodies were purchased from Sigma, Santa Cruz (Santa Cruz, CA) or Abcam (Cambridge, MA). Purified mouse IgG_2B_ and IgG_1_ myeloma protein were from Cappel Pharmaceuticals, Inc. TUNEL was obtained from Roche Ltd (Germany). All experiments were performed under the regulations of Rutgers University and the NIH.

### Microglia isolation

Human fetal CNS tissue was used as part of an ongoing research protocol approved by Rutgers University (IRB protocols Pro2012001303 and Pro20140000794). Microglia was established as previously described^97^. Briefly, the tissue was minced and shaken. The slurry was passed through a 250 µm nylon mesh filter followed by a 150 µm filter, washed once with HBSS, and then with complete DMEM (DMEM plus 25 mM HEPES, 10% FCS, 1% penicillin-streptomycin, 1% non-essential amino acids). Cells were resuspended and seeded at 9 × 10^7^ per 150 cm^2^ flask for 12 days. The medium, containing microglia, was then removed and centrifuged for 5 minutes at 220 × g. The microglia was resuspended in complete DMEM and seeded according to the experiment required. Microglia were infected with HIV and medium was collected every 3 days to measure HIV replication. All experiments performed in microglia were determining HIV-replication as determined by ELISA and apoptosis or survival as determined by microscopy. The main limitation of these cultures is the numbers of microglia.

### Monocyte isolation and macrophage culture

Human monocytes were isolated from leukopaks obtained from the New York Blood Center. Peripheral blood mononuclear cells (PBMCs) were isolated by differential centrifugation using a Ficoll gradient (GE Healthcare, Piscataway, NJ). Adherent cells were cultured for 7 days in the presence of 10 ng/ml macrophage colony stimulating factor (Miltenyl Biotec, San Diego, CA) in RPMI 1640 with 10% FBS, 5% human AB serum, 1% P/S, and ten mM HEPES to differentiate the cells into macrophages.

### HIV infection and replication

After seven days in culture to enable differentiation, macrophages were inoculated with 20–50 ng/ml HIV_ADA_ for 24 hours, and then apoptosis, fusion, and expression of apoptotic proteins was examined. Supernatants were collected, and the medium was changed every 24 hours until 7, 14, 21 and 28 days post-inoculation. Viral replication was analyzed by HIV p24 ELISA according to the manufacturer’s instructions.

### Live cell imaging

To assess fusion, macrophages were imaged using a Zeiss AxioObserver Z1 with an LD Plan-Neofluar 5X/0.4 10x air objective lens and a Zeiss AxioCam MRm camera using Axiovision software. Stage and objectives were housed within an incubation chamber maintained at 37 °C and 5% CO_2_.

### HIV integration by fluorescent in situ hybridization (FISH)

Uninfected and HIV-infected macrophages were placed in an ultra-clean glass and incubated in probes directed to HIV-Nef DNA, and Alu repeats as well as immunofluorescence for Iba-1, a macrophage marker, and actin as well as DAPI to label all the nuclei. The resolution of our equipment corresponds to 20 nm per pixel. Thus, colocalization of DAPI, HIV-Nef DNA, and DNA-Alu correspond to integrated HIV DNA. This staining method enables us to detect few copies (even a single copy of HIV integrated DNA) of integrated DNA in the host DNA, however, in this manuscript we did not quantify copy numbers, we only quantified negative versus positive cells. Thus, we expect perfect colocalization between DAPI, Alu repeats, and the HIV integrated DNA is HIV DNA is inserted into the host DNA.

### Alu-gag PCR

Integration of HIV into the host genome was detected by Alu-gag PCR as described previously with minor variations^98^. The system was calibrated using OM-10 cells and diluted OM-10 cells into millions of uninfected Hela cells to quantify the lowest numbers of copies possible in 10^8^ to 10^12^ cells.

### *In situ* Gag RNA analysis

To examine the formation and localization of gag mRNA, RNAscope was used (Newark, CA). However, we analyzed the data using fluorescence because localization of the RNA is more informative than by colorimetric analysis. We used the same protocol described by the provider in combination with immunofluorescence to detect DAPI (to label nuclei) and Iba-1 (a macrophage marker) in the same cell.

### Immunofluorescence

Human macrophages, HIV-infected and uninfected, were grown on glass coverslips, fixed and permeabilized in 70% ethanol for 20 min at −20 °C or fixed in 4% paraformaldehyde and permeabilized with 0.01% Triton-X for 2 minutes. Cells were incubated in TUNEL reaction mixture (Roche, Germany) at 37 °C for 1 h, washed three times with PBS and incubated in blocking solution for 30 min at room temperature. Cells were incubated in blocking solution for 30 min at room temperature and then in primary antibody (anti-HIV-p24 or isotype controls: both 1:50) overnight at 4 °C. Cells were washed several times with PBS at room temperature and incubated with phalloidin conjugated to Alexa Fluor 488 (Thermo-Fisher, Carlsbad, CA) to identify actin filaments and/or the appropriate secondary antibody conjugated to FITC (Sigma, St. Louis, MO) for 1 h at room temperature, followed by another wash in PBS for 1 h. Then, cells were mounted using anti-fade reagent with DAPI. Cells were examined by confocal microscopy using an A_1_ Nikon (Tokio, Japan) to quantify the total numbers of cells as well as TUNEL positive cells.

### Western Blot Analysis

Samples were lysed with RIPA buffer (Cell Signaling, Beverly, MA) containing protease inhibitors (Cell Signaling, Danvers, MA), and 50–80 µg of protein were electrophoresed on a 4–20% polyacrylamide gel (Bio-Rad), and transferred to nitrocellulose membranes. Membranes were probed with the mitochondrial antibodies described and developed with HRP (see original blots in Supplemental Fig. 1). Densitometric analysis was performed using NIH ImageJ software. The original gels are presented in supplemental figure 1.

### Human tissue sections

Human tissues were collected as part of the IRB-approved for Rutgers University and the Manhattan HIV Brain Bank and National NeuroAIDS Tissue Consortium (NNTC). Sections of 15 to 25 µm thickness were processed for immunofluorescence and confocal microscopy as described above (n = 11, four uninfected and five HIV-infected with no viral replication detected for 6–24 years, see Table 1 for details).

**Table 1:** Patient Information.

Patient number	HIV status	Age	Gender	ART Current ARVs (5 y of death)	CD4 counts (cells/mm)	Viral Load (copies/ml)	Years with HIV
1	−	42	M	N.A.	N.D.	N.A.	N.A.
2	−	45	F	N.A.	N.D.	N.A.	N.A.
3	−	38	F	N.A.	N.D.	N.A.	N.A.
4	−	42	F	N.A.	N.A.	N.A.	N.A.
5	+	31	M	3TC, D4T	241	17226	24 y
6	+	50	F	CBV, KTA, TFV	111	<50	24 y
7	+	41	M	ATV, RTV, TRU	228	<50	11 y
8	+	48	M	ATV, DDI, TFV	265	181	6 y
9	+	48	M	3TC, EFV, TFV	255	<50	13 y

Notes: M: male; F: Female; N.A.: not applicable; N.D.: not determined.

Anti-retrovirals: 3TC, lamivudine (Epivir); ATV, atazanavir (Reyataz); CBV, zidovudine + lamivudine (Combivir); DDI, didanosine (Videx); KTA, LPV/RTV; lopinavir/ritonavir (Kaletra); RTV, ritonavir (Norvir); TFV, TFV, PMPA; tenofivir DF (Viread); DRV, TMC-114, darunavir (Prezista); TRU, emtricitabine + tenofovir (Truvada).

### Statistical analysis

Statistical analyses were used to determine the significance of data from all experiments. Significance was assessed by determining the validity of the null hypothesis that states that all treated groups were the same as their respective controls or HIV infection alone. Origin 8 software was used to test the null hypothesis by comparing the relative value to a theoretical mean of 1 using a two-tailed, two sample t-test with a 95% confidence intervale.

## Electronic supplementary material

Original Western blots
