# Supplementary material for: HIV-infected macrophages and microglia that survive acute infection become viral reservoirs by a mechanism involving Bim
Source: Sci Rep. 2017 Oct 9;7:12866. doi: 10.1038/s41598-017-12758-w (PMC5634422; doi:10.1038/s41598-017-12758-w)

HIV-infected macrophages and microglia that survive acute infection become viral reservoirs by a mechanism involving Bim

Castellano Paul, Prevedel Lisa., and Eliseo A. Eugenin

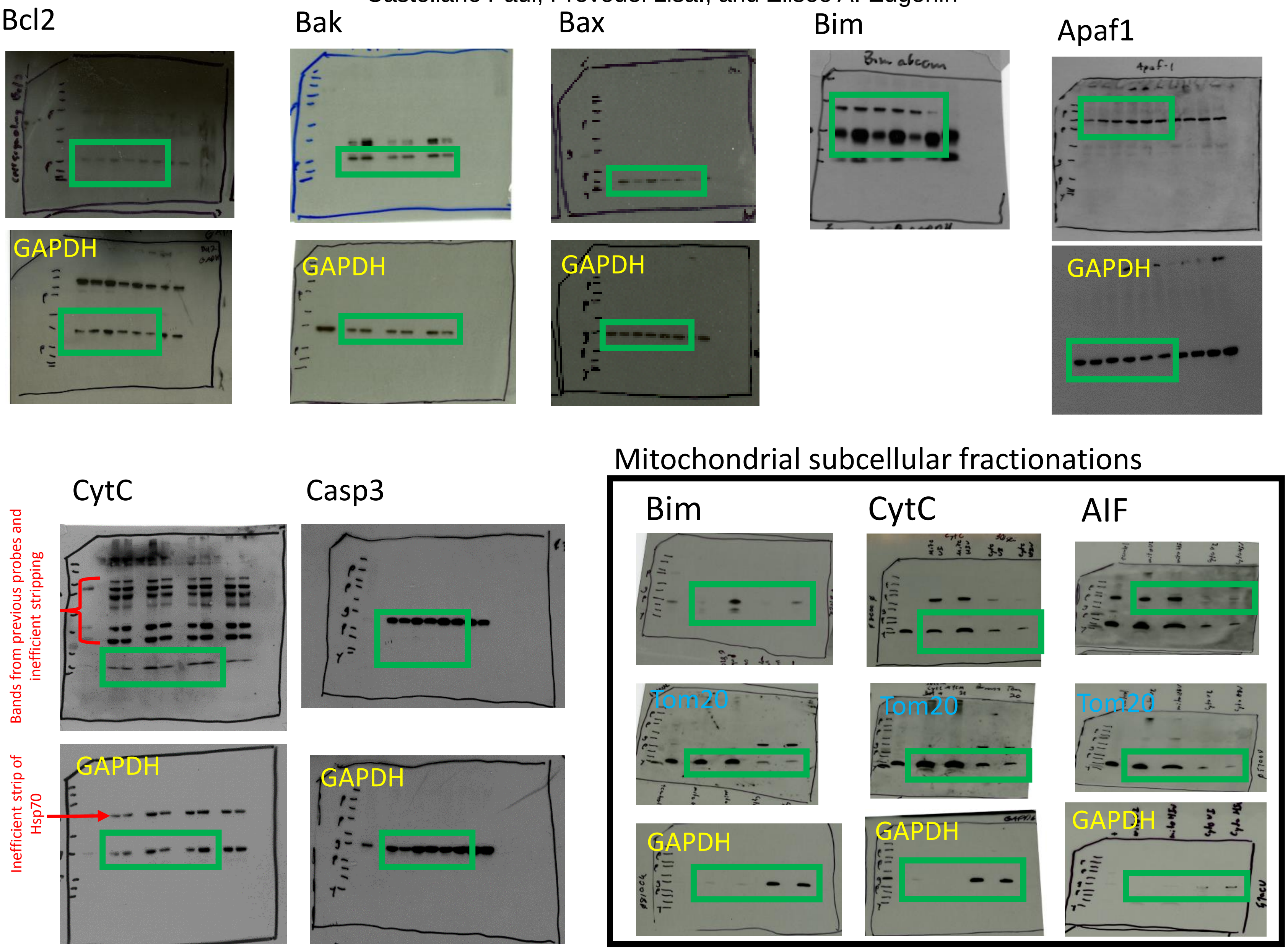

Supplement: Supplementary file 1 — Original Western blots [file 41598_2017_12758_MOESM1_ESM.pdf]
